# Protein array profiling of circulating angiogenesis-related factors during bevacizumab containing treatment in metastatic colorectal cancer

**DOI:** 10.1371/journal.pone.0209838

**Published:** 2018-12-28

**Authors:** Helga Hagman, Pär-Ola Bendahl, Jon Lidfeldt, Mattias Belting, Anders Johnsson

**Affiliations:** 1 Department of Clinical Sciences Lund, Section of Oncology and Pathology, Lund University, Lund, Sweden; 2 Department of Oncology, Skåne University Hospital, Lund, Sweden; University of South Alabama Mitchell Cancer Institute, UNITED STATES

## Abstract

**Background:**

Prolonged angiogenesis inhibition may improve treatment outcome in metastatic colorectal cancer (mCRC) patients. However, due to the complexity of the angiogenic pathways there is a lack of valid predictive biomarkers for anti-angiogenic agents. Here, we describe and optimize a procedure for simultaneous dynamic profiling of multiple angiogenesis related proteins in patient serum to explore associations with the response and acquired resistance to anti-angiogenic therapy.

**Materials and methods:**

Patients (n=22) were selected from a clinical trial investigating maintenance treatment with bevacizumab alone after response to induction chemotherapy + bevacizumab in mCRC. Serum samples were analysed for 55 unique angiogenesis related proteins using a commercial proteome profiler array and a publicly available image analysis program for quantification. Samples were collected at baseline before induction treatment start, at start of maintenance treatment, and at end of treatment after tumour progression.

**Main results and conclusion:**

For eight proteins, the antibody array signals were below detection range in all patient samples. None of the proteins showed levels at baseline or at start of maintenance with strong evidence for correlation to time to progression (lowest nominal p-value 0.03). The dynamic ranges of protein levels measured during the induction treatment period and during the maintenance period were analysed separately for time trends. Evidence for changing trends (up/down) in the levels of MMP-8, TIMP-4 and EGF was observed both during response to induction treatment and at progressive disease, respectively. For three of the proteins (IL-8, Activin A and IGFBP-2), weak evidence for correlation between increasing protein levels during induction with chemotherapy and bevacizumab and time to progression was observed.

In conclusion, semi-quantitative profiling of angiogenesis related proteins in patient serum may be a versatile tool to screen for protein patterns aiming at identifying resistance mechanisms of anti-angiogenic treatment in patients with mCRC.

## Introduction

Over four decades ago angiogenesis inhibition was proposed as a strategy to treat cancer [1], and since then several therapeutic compounds targeting angiogenesis have been introduced. These agents, which have played an important role in both oncological research and clinical practice, include small molecule tyrosine kinase inhibitors and antibodies targeting *e*.*g*. the vascular endothelial growth factor (VEGF) receptors and its ligands. The first VEGF-inhibiting agent to be approved for treatment of solid tumours was bevacizumab, which is a monoclonal antibody that binds to circulating VEGF-A [2]. In metastatic colorectal cancer (mCRC) several randomized trials have demonstrated survival gain in patients receiving bevacizumab combined with chemotherapy compared to chemotherapy alone [3]. Some patients also benefit by a prolonged inhibition of angiogenesis in the continuum of care, as demonstrated in mCRC trials by anti-angiogenic antibody addition to chemotherapy beyond progression [4–6]. Bevacizumab continuation is also applied in maintenance treatment strategies as an alternative to treatment break after response on induction chemotherapy plus bevacizumab in different metastatic tumour settings [7, 8].

In mCRC, maintenance treatment with bevacizumab and a fluoropyrimidine has shown a significant improvement in progression free survival (PFS) but not in overall survival (OS) [9], whereas bevacizumab alone as maintenance treatment only leads to a very modest prolongation of PFS compared to complete treatment break [10].

Accordingly, the use of single bevacizumab is not recommended in mCRC [10, 11], since the lack of predictive biomarkers prevents clinicians from selecting patients with the best chance to respond to this strategy.

VEGF-A is known as the main regulator of tumour angiogenesis [12, 13]. However, cancer angiogenesis is a complex process involving multiple receptors, ligands and intracellular proteins produced by tumours and the host. These pro- and anti-angiogenic proteins, here referred to as angiogenesis related factors, can be detected in the circulation [12]. In search for predictive biomarkers and increased mechanistic understanding of the effects and resistance of VEGF-inhibition, the use of multiplex protein assays has many advantages [14, 15] and include different techniques that have been applied in previous reports in mCRC [16–21].

In the present study, we explore a commercially available antibody protein array for the simultaneous assessment of 55 different angiogenesis related proteins in serially collected serum samples from mCRC patients selected from a randomized clinical trial investigating induction chemotherapy with bevacizumab followed by maintenance single bevacizumab until tumour progression. The primary aim was to study the utility of this protein assay on frozen serum samples by optimizing experimental conditions using conventional immunoblotting methodology integrated with readily available image analysis software. With this procedure, we explore possible relations between the protein patterns and clinical outcome, in terms of time to tumour progression (TTP), and search for predictive biomarkers of bevacizumab maintenance treatment. Furthermore, we investigate changes in circulating protein levels at the time of progression during treatment with bevacizumab alone, which could provide new insights into acquired resistance mechanisms.

## Materials and methods

### Patients

**Fig 1:**
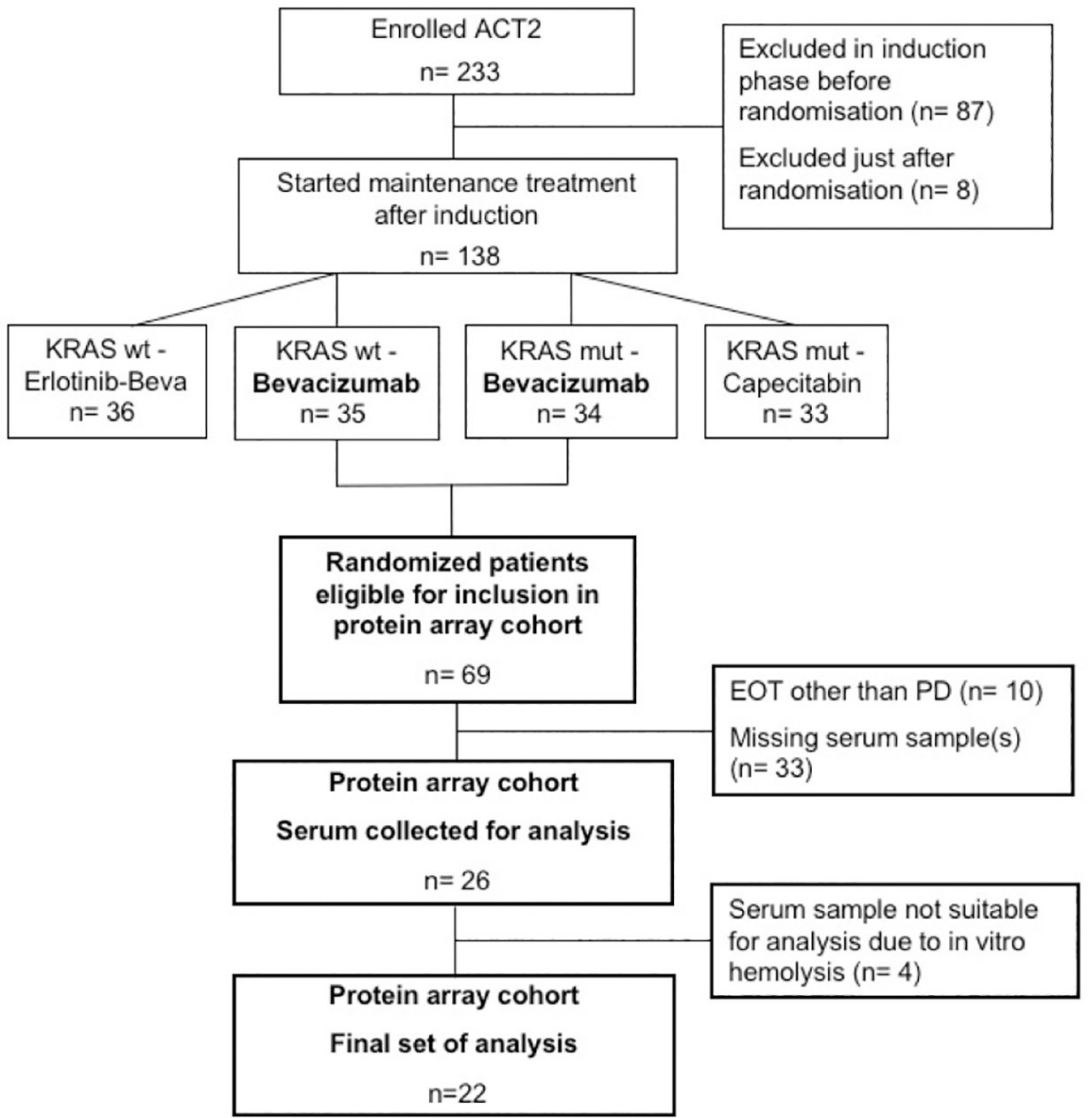
Patient flow chart. The induction treatment phase of the ACT2 trial consisted of chemotherapy plus bevacizumab for 18 weeks, and was followed by randomisation to maintenance treatment. KRAS, Kirsten Rat Sarcoma Viral Oncogene; wt, wild-type; mut, mutated; EOT, End of Treatment; PD, Progressive Disease.

Patients were selected from the randomized clinical trial Nordic ACT2 (ClinicalTrials.gov: NCT01229813)[22], including patients with untreated mCRC, performance status ECOG 0-1, adequate organ function and no evidence of significant active cardiovascular disease or uncontrolled hypertension. The main purpose of the ACT2 trial was to investigate different maintenance treatment strategies after response to first line induction treatment. For the present study, we selected patients that had at least stable disease on induction treatment, who were randomized to single bevacizumab maintenance treatment, and who had progressive disease (PD) as reason for end of treatment (EOT) (Fig 1). Another prerequisite for inclusion was that serum samples were available from all three pre-defined time-points (see below). This study was conducted according to the Declaration of Helsinki and was approved by the Regional Ethics comittee of Lund (2010/129). All participating subjects signed written informed consent to participate in the study.

### Anti-tumoral treatment regimens

In the ACT2 trial induction treatment was given with 5-fluorouracil (5-FU) or capecitabine in combination with oxaliplatin or irinotecan (FOLFOX/ FOLFIRI or XELOX/ XELIRI) plus bevacizumab, 5 mg/kg intravenously biweekly or 7.5 mg/kg every third week depending on chemotherapy schedule. In the absence of PD at second tumour evaluation after 18 weeks, patients were eligible for randomization to maintenance treatment, guided by Kirsten rat sarcoma oncogene (KRAS) status (S1 Fig). Both patients with KRAS wild-type and mutated tumours could be randomised to either of the two arms that included single bevacizumab treatment (7.5 mg/kg every three weeks), *i*.*e*. arms wt-B and mut-B, from which we retrieved patients for the present study (Fig 1)

### Tumour evaluation and blood sampling

Response evaluation was performed with a computed tomography (CT) scan of the thorax and abdomen according to RECIST 1.0 criteria at baseline and twice during induction treatment. Patients with objective tumour response (stable disease, SD or partial response, PR) at 18 weeks, were randomised to maintenance and tumour evaluation was repeated every nine weeks during the maintenance phase until evidence of PD.

Venous blood samples for translational research were collected from each patient on three occasions defined in the protocol: 1) at baseline (BL), i.e. on the day of or at maximum 7 days before treatment start, 2) prior to start of maintenance (SOM), i.e. at first cycle of single bevacizumab after randomization, and 3) at off study visit within 30 days from the final bevacizumab cycle, i.e. at date of end of treatment due to PD (EOT) (S1 Fig). At each occasion 5 ml of blood was collected in a serum tube which was put to rest in room temperature for 30 minutes and then centrifuged at 2000 x g for 10 minutes. Serum was aliquoted into one 1.5 mL cryovial and stored at -70° C for later analyses.

### Protein array analysis

Angiogenesis related proteins were analysed using the Proteome Profiler Human Angiogenesis Antibody Array (R&D Systems, Minneapolis, MN, USA), which allows simultaneous analysis of 55 proteins (soluble growth and differentiation factors, extracellular matrix components, proteases, membrane-bound receptors, and intracellular signalling molecules), shown in Table 1. The antibody array is a membrane based sandwich immunoassay using chemiluminescence for visualization according to standard immunoblotting procedures. The preparation and incubation of the arrays were performed as recommended by the manufacturer, and as previously reported [23]. To optimise the exposure time, allowing the detection and pixel quantification of a maximum number of protein spots, we initially tested six different exposure times, ranging from 30 s to 60 min, for each membrane. In the final analyses, we decided to use the 3 min and 30 min exposures (see Results). The developed array films were scanned, size adjusted, inverted in Adobe Photoshop, overlaid with a template and imported into Image J (https://imagej.nih.gov/lj) for quantification of the pixel intensity in each spot (S1 Fig). An average signal was calculated of the duplicate spots representing each protein, followed by background subtraction of a clear area of the membrane. Finally, each protein specific signal was divided by the average signal of the six positive reference spots of the membrane. The resulting normalized ratios, henceforward referred to as protein levels, were used in the subsequent analyses.

**Table 1:** Detection of angiogenic factors in the protein array patient cohort (n=22 patients).

Protein	Alternative nomenclature	Detectability (% of patients)		
		BL	SOM	EOT
Activin A		18	14	14
ADAMTS-1		ND	5	5
Amphiregulin	AR, AREG	14	5	18
Angiogenin	ANG, ribonuclease 5	100	100	100
Angiopoietin-1	Ang-1	95	100	95
Angiopoietin-2	Ang-2	9	5	9
Angiostatin	Plasminogen	95	95	95
Artemin	Enovin, Neublastin	9	5	5
Coagulation Factor III	TF, tissue factor, thromboplastin, CD142	14	9	18
CXCL16	Chemokine (C-X-C motif) ligand	100	95	95
DPPIV	CD 26	100	100	95
EG-VEGF	PK1	10	5	9
EGF	Endothelial Growth Factor	71	18	36
Endoglin	CD105	100	100	95
Endostatin	Collagen XVIII	100	95	95
Endothelin-1	ET-1 +	81	68	82
FGF acidic	Fibroblast Growth Factor, FGF-1	5	ND	5
FGF basic	FGF-2, FGF-β	ND	ND	ND
FGF-4	Fibroblast Growth Factor, FGF-4	5	5	ND
FGF-7	KGF Keratinocyte growth factor	ND	ND	ND
GDNF	Glial Cell Line-derived Neurotrophic Factor	ND	ND	ND
GM-CSF	Macrophage colony stimulating factor	5	9	9
HB-EGF	Heparine binding EGF like growth factor	64	59	50
HGF	Hepatopoietin A /Scatterfactor	77	55	73
IGFBP-1	IGF-binding protein	100	100	100
IGFBP-2	IGF-binding protein	100	100	100
IGFBP-3	IGF-binding protein	95	95	95
IL-1β	Interleukin-1 beta, IL-1F2	9	ND	5
IL-8	Interleukin-8, CXCL8	14	5	9
LAP/TGF-β1	Latency Associated Peptide	23	18	18
Leptin		91	100	100
MCP-1	Monocyte chemotactic protein-1, CCL2	ND	ND	ND
MIP-1α	CCL3.Macrophage Inflammatory Protein.	ND	ND	ND
MMP-8	Matrix Metalloproteinase- 8	100	100	100
MMP-9	Gelatinase B	95	95	95
NRG1-β1	HRG1-β1 Neuroregulin/Heregulin-1 β	9	5	5
PD-ECGF	TYMP	23	23	23
PDGF-AA	Platelet Derived Growth Factor AA	100	100	100
PDGF-AB/PDGF-BB	Platelet Derived Growth Factor AB/BB	91	86	86
Pentraxin 3 (PTX3)	TSG-14	100	100	100
Persephin		14	9	9
Platelet Factor 4	PF4, CXCL4	95	95	91
PlGF	Placenta Growth Factor	14	18	18
Prolactin		100	100	100
Serpin B5	Maspin	ND	ND	ND
Serpin E1	PAI-1	95	95	95
Serpin F1	PEDF	32	32	27
Thrombospondin-1	TSP-1	77	86	86
Thrombospondin-2	TSP-2	23	14	14
TIMP-1	Tissue Metalloproteinase Inhibitor 1	100	100	100
TIMP-4	Tissue Metalloproteinase Inhibitor 4	100	100	100
uPA	urokinase-type Plasminogen Activator	91	91	86
Vasohibin		ND	ND	ND
VEGF	Vascular Endothelial Growth Factor	91	100	95
VEGF-C		ND	ND	ND

Proteins analysed by the Human angiogenesis antibody array (n=55), in alphabetical order. Serum samples collected at BL, baseline before treatment start; SOM, start of maintenance, after induction chemotherapy + bevacizumab before first cycle of bevacizumab maintenance and at EOT, End of treatment, after last cycle of bevacizumab at progression on maintenance therapy. ND, protein not detectable in any patient sample (0%).

### Statistical methods

Time to progression was defined as the time from start of treatment, i.e. the date of first treatment cycle in induction phase to the date of progressive disease on maintenance treatment (TTP1). The time from SOM, *i*.*e*. the date of first single bevacizumab maintenance treatment cycle, to the date of progressive disease was defined as TTP2. Associations between BL protein levels and TTP1 were, due to small sample size and skewed distributions, analysed using Spearman rank correlation. The reason for not using standard methods for survival analysis was that only patients ending treatment due to progression were included in the study. Associations between TTP2 and protein levels at SOM and/or relative changes in protein levels from BL to SOM were also evaluated using Spearman correlation in a landmark analysis.

For each protein, assessments below the detection limit were, instead of given a nil value, set to 50% of the lowest protein level measured in any patient at any of the three time points for that protein. Dynamic changes in log protein levels between two adjacent time point (BL and SOM, or SOM and EOT) were, also due to the small sample size and skewed distributions, evaluated using the Wilcoxon matched-pairs signed-ranks test. The reason for choosing the multiplicative log-scale was that the pixel intensity was expressed semi-quantitatively as arbitrary units for which absolute changes are not meaningful.

The p-values calculated for association to TTP and for dynamic changes have not been adjusted for multiple testing. Strict application of e.g. Bonferroni correction would, in the present study, have been too conservative due to information loss caused by frequent non-detectability. The level of evidence for a specific association, *i*.*e*. the p-value, should hence be interpreted with caution. The main purpose of the significance tests performed and presented in this exploratory study was to rank the proteins according to evidence. Throughout the paper, we use the term weak evidence for nominal p-values in the range 0.005 to 0.05. The statistics package Stata version 14.2 (StataCorp LP, 2016, College Station, TX, USA) was used for statistical calculations and graphics.

## Results

### Patient characteristics

Twenty-six patients fulfilled the inclusion criteria and were included in the intended analysis cohort, but four of these patients were excluded due to in vitro hemolysis in at least one of the three serum samples. Thus 22 patients were finally included in the study (Fig 1). The baseline patient characteristics of the cohort are presented in Table 2.

**Table 2:** Patient baseline characteristics of the protein array cohort (n=22).

Characteristic		No. of patients
Gender	M/F	12/10
Age	Median (range)	63 (44-75)
Performance Status	0/1	16/6
Induction chemotherapy regimen	XELOX/FOLFOX	10
	XELIRI/FOLFIRI	12
KRAS status	wildtype	9
	mutant	13
Response at end of induction treatment	SD/PR	11/11
Primary tumour location	colon	10
	rectum	11
	both	1
Hypertension	Yes/No	11/11
Diabetes	Yes/No	4/18
Cardiovascular disease	Yes/No	3/19
Thromboembolism	Yes/No	0/22

M= Male, F= Female. Performance status according to ECOG/WHO. KRAS = Kirsten rat sarcoma viral antigen.

### Methodological aspects

We initially performed a pilot analysis to test varying volumes of serum collected from a healthy, male control subject. The results showed a highly reproducible and robust pattern of protein signals, and a serum volume of 0,25 ml was found optimal for use in the subsequent patient sample analyses. For each protein membrane, six different exposure times to the x-ray film were used, as described in the Methods section. After visual inspection of developed membranes, we decided to use the three and 30 min exposure times for further analyses (S1 Fig). The reason for not using longer exposure times was the oversaturation of reference spots that precluded further normalization and quantification. We observed large differences in intensity levels between proteins, possibly reflecting that some proteins are more abundant than others in serum. However, epitope specificity and affinity of assay antibodies may also be of importance. As expected, the signals were higher after long exposure (30 min) than after short exposure (3 min) time. From the data presented in S2 Fig, we conclude that some low-abundant proteins with levels below the detection limit after short exposure could be reliably quantitated after the longer exposure. Further, for very high-abundant proteins the difference in pixel intensity between exposure times seemed to decrease, showing saturation at the maximum intensity level (set at 110 arbitrary pixel units). To optimise the possibilities for quantitative comparisons between samples, we defined and applied the following rule for selection of array data: For the proteins that were detectable with the short exposure time at all three time points (BL, SOM and EOT) in a specific patient, data from the membrane with short exposure was used in the analyses. In all other proteins, data from the membrane with long exposure time were used for further analyses.

### Protein detectability

In Table 1, all array specific proteins (n=55) are listed with the corresponding detection rate in the cohort at the analysis time points, BL, SOM and EOT. Eight of the proteins were not detectable in any of the analysed serum samples: FGF basic, FGF-7, GDNF, MCP-1, MIP-1α, Serpin B5, Vasohibin and VEGF-C (Table 1).

At the other end of the spectrum of signal intensity, detectable signals were seen in all the serum samples for Angiogenin, IGFBP-1, IGFBP-2, MMP-8, PDGF-AA, PTX3, Prolactin, TIMP-1, and TIMP-4. The proportion of proteins that were detectable in all patients at BL, SOM and EOT were 22%, 25%, and 18%, respectively. It is notable that among the proteins under detection level, most are growth factors and cytokines generally found at pg/ml levels in serum or plasma whereas in the high signal category, majority of proteins are chaperone proteins, proteases and protease inhibitors.

### Changes in protein levels

**Fig 2:**
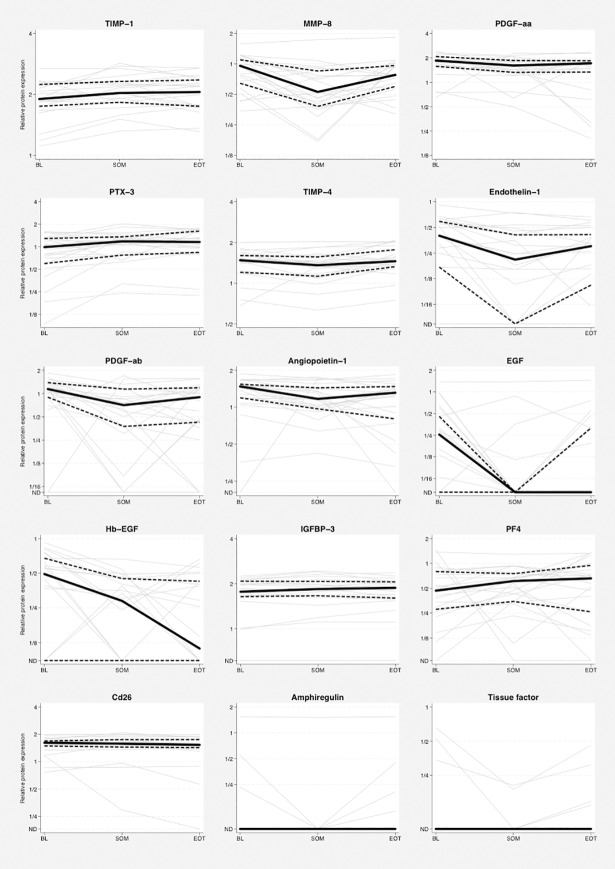
Proteins with evidence for changes in serial analysis (n=22 patients). Dynamics of log protein levels for the 15 proteins with nominal p-values <0.05 for change either from BL to SOM or from SOM to EOT. Faded grey lines connect relative protein levels for each patient. Black solid lines represent median values and dotted lines indicate upper and lower quartiles. Relative protein levels below the detection limit were set to 50% of the lowest registered value for each protein, respectively, and the corresponding label on the y-axis has been set to ND (Not Detected). Note that the lower quartile will be ND if at least 25% of the patients have values below the detection limit, the median will be ND if at least 50% of the patients have values below detection limit and that the upper quartile will be ND if at least 75% of the patients have undetectable levels of a protein. BL, baseline; SOM, start of maintenance treatment; EOT, end of treatment.

Between BL and SOM, *e*.*g*. during the induction phase with chemotherapy and bevacizumab, the levels of eight proteins (Endothelin-1, MMP-8, PDGF-ab, PDGF-aa, Angiopoietin-1, EGF, Hb-EGF, and TIMP-4) showed at least weak evidence for a decrease (nominal p-values <0.05). Four proteins increased (TIMP-1, PTX-3, IGFBP-3 and PF-4) during the same period (Table 3 and Fig 2). From SOM to EOT, e.g. during tumor progression on single bevacizumab treatment, weak evidence was observed for increase in the level of five proteins (TIMP-4, MMP-8, EGF, Amphiregulin, and Tissue factor/Coagulation factor III), whereas a decrease was seen in only one protein (CD26). Regarding VEGF-A, *i*.*e*. the target of bevacizumab, no evidence for changes were observed between the different time points.

**Table 3:** Proteins with nominal evidence for change (unadjusted p<0.05) during treatment of the protein array cohort (n=22 patients).

**A**					
**Protein**	**Dynamics of protein level (SOM – BL)** **(No. of patients)**			**Direction**	**p-value**
	**Increased**	**ND**	**Decreased**	**Up/down**	
TIMP-1	17	0	5	↑	0.004
PTX-3	16	0	6	↑	0.014
IGFBP-3	12	1	9	↑	0.044
PF-4	15	0	7	↑	0.046
Endothelin-1	3	5	14	↓	0.002
MMP-8	6	0	16	↓	0.004
PDGF-ab	2	1	19	↓	0.004
PDGF-aa	5	0	17	↓	0.006
Angiopoietin-1	3	0	19	↓	0.006
EGF	3	6	13	↓	0.006
Hb-EGF	2	6	14	↓	0.009
TIMP-4	7	0	15	↓	0.046
**B**					
**Protein**	**Dynamics of protein level (EOT – SOM)** **(No. of patients)**			**Direction**	**p-value**
	**Increased**	**ND**	**Decreased**	**Up/down**	
TIMP-4	16	0	6	↑	0.007
MMP-8	17	0	5	↑	0.019
EGF	7	14	1	↑	0.034
Amphiregulin	4	18	0	↑	0.046
Tissue factor	4	18	0	↑	0.046
Cd26	7	0	15	↓	0.017

**3A.** Changes from baseline (BL) to start of maintenance (SOM); Chemotherapy + Bevacizumab treatment. **3B.** Changes from start of maintenance (SOM) to End of Treatment (EOT); Bevacizumab treatment. ND= non-detectable serum protein levels.

### Protein levels and association with time to progression

The median TTP1 (BL to EOT) was 253 days (range 182-490) and median TTP2 (SOM to EOT) was 119 days (range 50-364), *i*.*e*. the average difference between TTP1 and TTP2 was 134 days (range 118-163). This corresponds to an average length of 19 weeks (4.7 months) from start of induction phase to start of maintenance treatment, well in line with the predefined schedules in the ACT2 protocol [22].

There were no strong correlations between BL levels of any of the proteins and TTP1. Nor did we find any associations between protein levels at SOM and TTP2 (data not shown).

### Dynamic changes of protein levels and association with TTP

When analyzing the change in protein levels from BL to SOM in relation to TTP2, weak evidence for correlations were detected by landmark analysis for three proteins: IL-8 (r_s_=0.53, p=0.011), IGFBP-2 (r_s_=0.43, p=0.047) and Activin A (r_s_=0.43, p=0.045). Thus, increasing levels during the induction treatment were associated with longer TTP2 for these proteins. It should be noted that IL-8 was detectable in only three patients, and Activin A in four patients at BL, whereas IGFBP-2 was detectable in all patients at all time-points (Table 1).

## Discussion

Considerable effort is focused on the identification of predictive biomarkers of oncological treatment. For angiogenesis inhibitors, such as bevacizumab, no predictive biomarkers have been validated, despite a well-defined target [13]. This reflects the multifactorial nature of the angiogenic process and that the effect of VEGF blockade is influenced by adaptive events that occur under the selection pressure of tumor vessel regression, triggered by *e*.*g*. aggravated hypoxia. Consequently, it is critical to monitor the dynamic changes imposed by angiogenesis inhibition to better predict treatment response and to understand the biology of resistance to these treatments. Here, we demonstrate the feasibility of using an antibody membrane array to simultaneously analyze serially sampled serum from patients with advanced CRC. In this exploratory study of a limited number of patients responding to first line induction treatment with chemotherapy and bevacizumab, and progressing on bevacizumab single maintenance treatment, we found evidence (nominal p-values <0.05) for changes in twelve of 55 studied angiogenesis related proteins during response, and in six of the proteins during progression. No strong associations between TTP and BL levels of any of the proteins were detected, whereas weak evidence for correlation between raising levels of three proteins during induction therapy and longer TTP was observed.

For analyses of multiple serum proteins as predictive biomarkers, previously published studies in mCRC, have used different techniques, such as magnetic antibody-conjugated beads [16, 18, 21] and multiplex ELISA [19, 20], whereas Bai and colleagues [17] used a membrane-based antibody array based on the sandwich-ELISA principle, similar to the one used in the present study. An advantage with membrane based assays is that they are based on standard laboratory equipment available in most clinical research laboratories, and that image analysis and quantification can be performed in a standardized manner with an easily accessed software (here, Image J). This allows rapid analyses of multiple proteins in serum samples of limited volume, thus offering a convenient method for use in the clinical research setting. A disadvantage is that the measurements are semi-quantitative, *i*.*e*. the absolute concentrations of the proteins are not retrieved, which should pose a minor problem when comparing serial samples for assessment of dynamic events during treatment.

Another issue that became apparent from our results, is the low sensitivity for the detection of some angiogenesis related proteins in serum. One third of the measured proteins was detected in <10%, *i*.*e*. in maximum 2 of the 22 patients. One obvious reason for this is that the concentration of some proteins is very low in serum, but there may also be methodological explanations, such as antibody cross-reactivity or protein-complex interactions that conceal the protein epitopes from the membrane-bound capture antibodies or from the soluble detection antibodies [14]. Many of the proteins we studied, *e*.*g*. VEGF-A, occur in different splice variants [12], which could generate varying detectability depending on the assay used. Furthermore, some studies have reported a clear decrease in circulating VEGF-A after initiation of bevacizumab treatment [18, 24], whereas others, including our study, show no difference or even an increase in VEGF-A during bevacizumab treatment [25]. An explanation to the contradictions in the literature is that some assays measure free VEGF-A, whereas other methods also detect inactive VEGF-A bound to the antibody [25, 26]. Accordingly, these discrepancies motivate further methodological studies to refine antibody-based analysis of angiogenesis related proteins.

The presented dynamic variations from BL to SOM, during response to induction treatment, showed decreasing levels of eight proteins and increasing levels in four proteins (Table 3). Interestingly, almost all proteins that decreased (MMP-8, Hb-EGF, Angiopoietin-1, Endothelin-1, PDGF-ab, PDGF-aa, and EGF) have mostly proangiogenic properties, whereas those with the most significant increase (TIMP-1 and PTX-3) are primarily anti-angiogenic. One may speculate that the inhibition of angiogenesis occurring during successful treatment with chemotherapy and bevacizumab is not limited to the blockage of VEGF-A, but includes collateral modulation of several other angiogenesis-related ligands, partly through co-regulatory mechanisms with VEGF signaling. Correspondingly, four of the five proteins that increased during maintenance therapy with bevacizumab have primarily proangiogenic properties (MMP-8, Amphiregulin, EGF and Tissue factor/Coagulation factor III). Since the EOT samples were collected by the time of tumor progression on maintenance bevacizumab, these proteins may be involved in the acquisition of resistance against bevacizumab and thus potentially interesting targets to tailor subsequent anti-angiogenic treatment. Although, it is difficult to separate any such associations from the general effects of tumor response and progressive disease, respectively, our observations may still be relevant from a biomarker perspective. The three proteins (MMP-8, TIMP-4 and EGF) that following a decrease during tumor inhibition also showed evidence for an increase during progression are involved in cancer cell survival, proliferation and migration [27].

One of the proteins with evidence for increase during response to induction treatment was PTX-3, which is an extracellular matrix associated molecule that like C-reactive protein (CRP) belongs to the pentraxin-family. PTX-3 inhibits the pro-angiogenic effects of Fibroblast Growth Factor-2 (FGF basic), and has low affinity for VEGF [28]. Interestingly, PTX-3 has been suggested as a novel biomarker of hypertension [29], which is a common toxic effect of bevacizumab that is associated with improvement of the anti-angiogenic treatment effect [30, 31]. In our small cohort, the increase in PTX-3 during chemotherapy plus bevacizumab was not associated with longer TTP on bevacizumab alone as maintenance, and it is possible that a rise in PTX-3 levels occurs in response to chemotherapy alone. Nevertheless, PTX-3 merits further investigation as a potential biomarker for treatment effect in patients receiving anti-VEGF treatment.

Patients with progressive disease during the induction chemotherapy-based treatment were not included in the present series. Therefore, we did not analyze the associations between protein levels and objective response according to RECIST. Instead, the clinical outcome was studied in terms of TTP. TTP1, which was defined as the time from start of induction therapy until progression, is affected by the efficacy of both the chemotherapy and bevacizumab, whereas TTP2, that was calculated from the start of maintenance treatment, better reflects the potential efficacy of continued maintenance with bevacizumab. A clinically useful biomarker should preferably be one that predicts the outcome before the initiation of a treatment, but as given above this has proven to be challenging with angiogenesis inhibitors. This notion is supported by our results, as we did not find any evidence for associations between protein levels at BL and TTP1 for any of the proteins, nor between protein levels at SOM and TTP2. Bai and colleagues showed that low baseline levels of VEGF-A, HGF and ANGPTL4 were significantly associated with longer PFS whereas a low baseline level of Activin A was associated with shorter PFS [17]. Kopetz *et*. *al* found that patients with elevated levels of IL-8 at baseline had a shorter PFS [19]. It should be noted though that no predictive biomarker for the efficacy of bevacizumab or other anti-VEGF treatments has yet been validated for use in clinical practice [13]. In the analysis of serial serum assays, we found that increasing levels from BL to SOM of three proteins (IL-8, IGFBP-2 and Activin A) correlated with longer TTP2, *i*.*e*. a prolonged effect of maintenance bevacizumab. The results of IL-8 and Activin A were based on very few patients whereas IGFBP-2 was detectible in all samples. IGFBP (IGF-binding Protein)-2 modulates the action of Insulin Growth factors (IGF-1 and 2) involving a system that contributes to the pathogenesis of CRC, and serum levels of IGFBP-2 are found to be elevated in CRC patients [32, 33]. Experimental studies have shown that IGFBP-2 can induce VEGF production and that IGF-2 activates hypoxia inducible factor-1 (HIF-1) which in turn may induce IGFBP-2 as well as VEGF expression [34, 35]. This proposed autocrine loop to promote angiogenesis and tumor progression is difficult to reconcile with our results showing that patients with increased IGFBP-2 during induction treatment seem to benefit from prolonged bevacizumab maintenance treatment.

One may speculate that patients with upregulated HIF-1 and IGFBP-2 pathways as a response to induction treatment have a tumor phenotype with maintained VEGF dependency and thus responsiveness to prolonged VEGF inhibition during maintenance. Most likely an up regulation of IGFBP-2 mirrors the hypoxic state of the tumor through HIF-1α, a master transcriptional regulator of the hypoxic response. Thus, the demonstrated increased secretion of IGFBP-2 may be interpreted as a surrogate marker of effective vascular regression. It has also been suggested that IGFBPs can act as negative regulators of IGF activity or influence tumor growth independent from IGFs [33]. Further studies are needed to clarify the role of the IGF cascades in relation to the effects of bevacizumab.

Recent randomized trials have shown that maintenance treatment with bevacizumab alone has a modest effect in unselected patients [8, 10, 36], and predictive biomarkers would be helpful to identify subgroups of patients that could benefit the most from this strategy. We therefore decided to perform the present study in patients exposed to maintenance treatment with bevacizumab alone, and we applied very strict inclusion criteria to get a well-defined population of patients. Only 22 patients fulfilled these requirements, which of course limits the possibilities to draw firm conclusions. However, the strengths of our study are that it is based on a prospective trial which increase the validity of the clinical data and that we investigated angiogenesis proteins before and after a strictly defined first line maintenance treatment with bevacizumab alone for mCRC. To our knowledge this is the first report of serial proteomic data during anti-angiogenesis treatment in this specific setting.

We conclude that the described membrane antibody array is a robust and user friendly platform for exploration of multiple angiogenesis related proteins in serum from patients with mCRC during bevacizumab-based treatment. In line with several previous reports we failed to identify any biomarkers in BL samples for the prediction of the anti-angiogenic treatment effect. The dynamic changes in three of the proteins during induction treatment correlated with the time to progression on maintenance treatment with bevacizumab alone, but the clinical significance of these findings is unclear. As a future perspective, we suggest the use of this method to evaluate multiple protein pattern signals in patients at the time of progression on bevacizumab based treatment. The mapping of alternative angiogenic pathways related to resistance of bevacizumab could be a useful tool for individualized anti-angiogenic treatment options in late line settings.

## Supporting information

S1 Fig Study design and method. I) Schematic design of the ACT2 trial including serum sampling. II) Protein array membranes exposed on chemiluminescence detection film for 3 min and 30 min respectively. Inversion of scanned images and application of template in the Image J software program. III) Detection of protein levels by selection and quantification of membrane spots, in the figure exemplified with referral to PDGF-aa.
(TIFF)

S2 Fig Pixel intensity after short (3 min) and long (30 min) exposure time of protein array membranes to detection film (n=22 patients). Raw data from all proteins in the patient samples from all time points (BL, SOM, EOT) are depicted and expressed as arbitrary units. BL, at baseline; SOM, at start of maintenance treatment; EOT, at end of treatment.
(TIFF)
