# Supplementary figures and images for: Protein array profiling of circulating angiogenesis-related factors during bevacizumab containing treatment in metastatic colorectal cancer
Source: PLoS One. 2018 Dec 28;13(12):e0209838. doi: 10.1371/journal.pone.0209838 (PMC6310295; doi:10.1371/journal.pone.0209838)

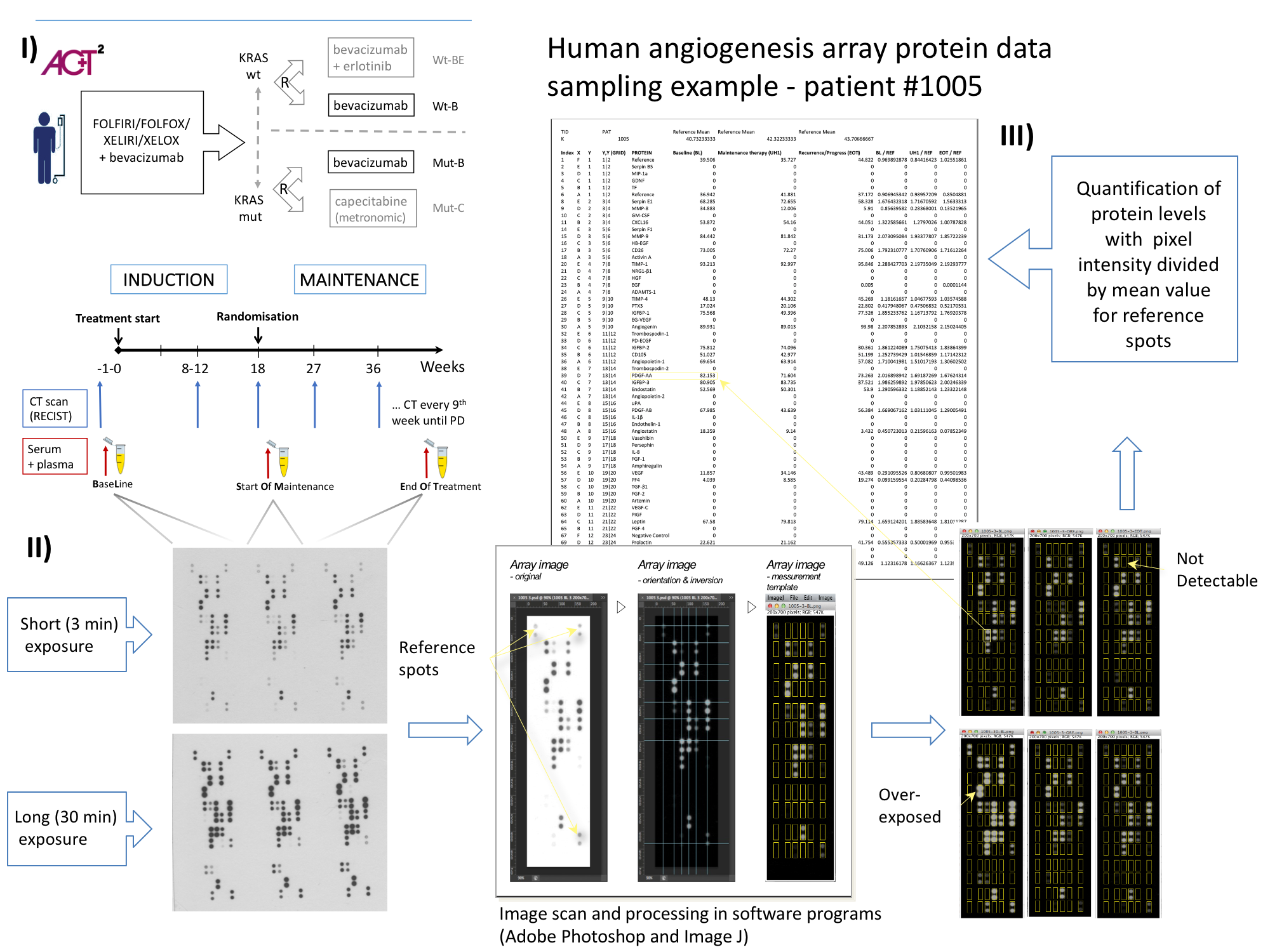

Supplement: S1 Fig — I) Schematic design of the ACT2 trial including serum sampling. II) Protein array membranes exposed on chemiluminescence detection film for 3 min and 30 min respectively. Inversion of scanned images and application of template in the Image J software program. III) Detection of protein levels by selection and quantification of membrane spots, in the figure exemplified with referral to PDGF-aa. (TIFF) [file pone.0209838.s001.tiff]

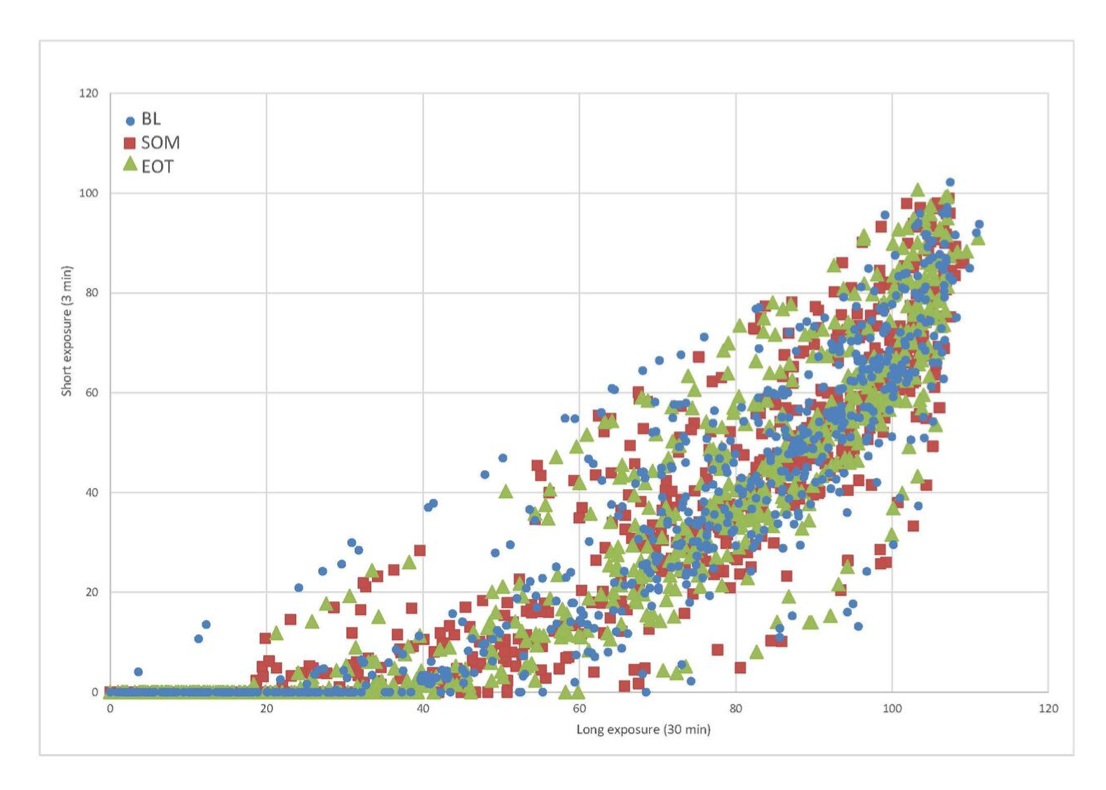

Supplement: S2 Fig — Raw data from all proteins in the patient samples from all time points (BL, SOM, EOT) are depicted and expressed as arbitrary units. BL, at baseline; SOM, at start of maintenance treatment; EOT, at end of treatment. (TIFF) [file pone.0209838.s002.tiff]
